# Hemodynamic stability with clonidine versus adrenaline in mandibular third molar surgeries

**DOI:** 10.6026/973206300211046

**Published:** 2025-05-31

**Authors:** Jyoti Bhavani Penumarti, Vinay Kharsan, Abhishek Balani, Abhishek Karan, Swati Sahu

**Affiliations:** 1 Department of Oral and Maxillofacial Surgery, New Horizon Dental College and Research Institute, Bilaspur, Chhattisgarh, India

**Keywords:** Clonidine, third molar surgery, local anesthesia

## Abstract

This randomized clinical trial compares 2% lignocaine with clonidine versus 2% lignocaine with adrenaline for impacted mandibular third molar surgery. Hence, 70 patients aged 18-40 were randomly allocated to either group. We evaluated hemodynamic stability, onset and duration of anesthesia. Clonidine showed lower intraoperative and postoperative systolic blood pressure (SBP), diastolic blood pressure (DBP), heart rate (HR) and mean arterial pressure (MAP). Though onset was slightly slower with clonidine, hemodynamic control was superior. Clonidine appears to be a safer alternative for patients requiring cardiovascular stability.

## Background:

Lignocaine hydrochloride is the most commonly used amide-type local anesthetic in dentistry due to its rapid onset and moderate duration of action [1]. To enhance the efficacy of local anesthetics, vasoconstrictors such as adrenaline are routinely added to prolong anesthetic duration and reduce systemic absorption [2]. However, adrenaline may induce undesirable cardiovascular effects such as tachycardia and elevated blood pressure, particularly in medically compromised patients [3]. Clonidine, an alpha-2 adrenergic agonist, has been investigated as a potential alternative vasoconstrictor due to its ability to produce sedation, analgesia and hypotensive effects without significantly stimulating the heart [4]. Studies have reported that clonidine enhances anesthetic duration and provides hemodynamic stability when used with lignocaine in dental and minor surgical procedures [5]. Despite several comparative studies, the clinical preference between clonidine and adrenaline in oral surgeries, particularly for impacted mandibular third molar removal, remains under investigation [6]. Therefore, it is of interest to report the comparative effects of clonidine and adrenaline as additives to lignocaine on hemodynamic parameters and anesthetic performance during the surgical removal of impacted mandibular third molars.

## Materials and Methods:

A randomized clinical study was conducted on 70 healthy patients (ASA I-II) aged 18-40 years requiring extraction of impacted mandibular third molars at the Department of Oral and Maxillofacial Surgery, New Horizon Dental College and Research Institute, Bilaspur, India. Ethical Approval: Ethical clearance was obtained from the Institutional Review Board (Approval No. NHDCRI/2022/MDS/OMFS/02-ECC).

## Study Design and Sample:

## Inclusion criteria:

[1] ASA I-II patients

[2] Age 18-40 years

[3] Minimal to moderate difficulty impactions (Pederson index)

[4] Interincisal mouth opening >30 mm

[5] Informed consent obtained

## Exclusion criteria:

[1] ASA III-IV patients

[2] Pregnant/lactating women

[3] Allergy to amide anesthetics, sulfites, or clonidine

[4] Local infections

[5] Previous dental procedure within 24 hours

Method of Preparation of Clonidine Solution: To prepare the anesthetic solution for Group A (Clonidine group), 9 ml of 2% lignocaine was drawn into a sterile 10 ml disposable syringe and 1 ml of clonidine hydrochloride solution (150 µg/ml) was added to it, resulting in a final concentration of 15 µg/ml clonidine with 2% lignocaine. The prepared solution was gently mixed and used within six hours. Any unused solution was discarded after six hours to maintain sterility and potency.

## Procedures:

Patients were randomly allocated into two groups:

[1] Group A (Clonidine Group): 2.5-3 ml of the prepared 2% lignocaine with 15 µg/ml clonidine was administered via Inferior Alveolar Nerve Block, Lingual Nerve Block and Long Buccal Nerve Block using a 26-gauge 25 mm disposable needle.

[2] Group B (Adrenaline Group): 2.5-3 ml of commercially available 2% lignocaine with 1:80,000 adrenaline was administered similarly via Inferior Alveolar Nerve Block, Lingual Nerve Block and Long Buccal Nerve Block using a 26-gauge 25 mm disposable needle.

Ward's incision and standard extraction techniques were used by a single surgeon. Hemodynamic parameters (SBP, DBP, HR and MAP) were recorded preoperatively, intraoperatively and postoperatively. Onset and duration of anesthesia and intensity of anesthesia (pain score), were measured.

## Statistical analysis:

Data were analysed using SPSS v24. Independent t-tests compared means. A p-value ≤ 0.05 was considered statistically significant.

## Results and Discussion:

A total of 70 patients were divided equally into Group A (clonidine) and Group B (adrenaline). Hemodynamic measurements showed consistent and statistically significant reductions in systolic blood pressure (SBP), diastolic blood pressure (DBP), heart rate (HR), and mean arterial pressure (MAP) in Group A throughout the perioperative period (p<0.05), highlighting the sympatholytic effect of clonidine (Figure 1 - see PDF, Table 1). Group A also demonstrated a statistically significant delay in onset of anesthesia (128.57 ± 13.43 sec) compared to Group B (112.37 ± 11.16 sec; p<0.001) (Figure 2 see PDF). While this delay was measurable, it did not impair clinical workflow or patient comfort, suggesting acceptable clinical utility. Additionally, the anesthesia duration in Group A (176.29 ± 18.88 min) was modestly shorter than in Group B (191.29 ± 19.03 min; p=0.001), without compromising surgical effectiveness (Figure 2 - see PDF, Table 1). This aligns with the known sympatholytic properties of clonidine. These findings align with those of Patel *et al.* [7], who also noted clonidine's favorable hemodynamic profile. Similarly, Alam *et al.* [8] and Souvik Chowdhury *et al.* [9] reported improved cardiovascular parameters when clonidine was used in dental surgeries. Tirupathi *et al.* [10] demonstrated comparable results, reinforcing the safety of clonidine in minor oral surgical procedures. In contrast, Brkovic *et al.* [11] observed that clonidine provided comparable anesthetic efficacy to adrenaline while maintaining greater hemodynamic stability, highlighting its clinical relevance in patients with cardiovascular concerns. In summary, although adrenaline provided a quicker onset and slightly extended anesthetic effect, clonidine ensured superior hemodynamic regulation. This makes it a compelling alternative in patients requiring cardiovascular vigilance during oral surgery.

## Conclusion:

Clonidine as an additive to lignocaine provides superior hemodynamic stability compared to adrenaline in mandibular third molar surgeries. Although onset is slightly delayed, clonidine maintains effective anesthesia with safer cardiovascular outcomes. This study supports its use as a safer alternative in medically sensitive patients.

## Funding:

No financial support was received for this study

## Author contributions:

All authors contributed equally to the conception, design and writing of the manuscript.

## Figures and Tables

**Table 1:** Comparative analysis of parameters between groups

**SBP (mmHg)**	**Preoperative**	**125.54 ± 10.44**	**118.46 ± 13.94**	**0.059**	**Not significant**
	Intraoperative	120.11 ± 10.62	126.43 ± 13.96	0.037	Significant
	Postoperative	119.66 ± 9.84	124.40 ± 13.44	0.046	significant
DBP (mmHg)	Preoperative	78.69 ± 9.10	75.43 ± 10.54	0.171	Not significant
	Intraoperative	74.40 ± 8.30	80.51 ± 10.99	0.011	Significant
	Postoperative	74.34 ± 7.30	78.66 ± 9.70	0.039	significant
HR (bpm)	Preoperative	79.71 ± 10.97	78.14 ± 7.90	0.494	Not significant
	Intraoperative	74.97 ± 9.70	83.03 ± 8.29	<0.001	Significant
	Postoperative	73.91 ± 8.92	80.89 ± 7.45	0.001	significant
MAP (mmHg)	Preoperative	94.18 ± 9.39	89.71 ± 11.49	0.08	Not significant
	Intraoperative	89.69 ± 8.76	95.69 ± 11.77	0.018	significant
	Postoperative	89.37 ± 7.81	94.15 ± 10.89	0.038	significant
Onset Time (sec)	N/A	128.57 ± 13.43	112.37 ± 11.16	<0.001	Significant
Duration (min)	N/A	176.29 ± 18.88	191.29 ± 19.03	0.001	Significant
